# Learning From the Uncommon in Common Practice: A Case Report on Metamizole-Induced Agranulocytosis

**DOI:** 10.7759/cureus.99539

**Published:** 2025-12-18

**Authors:** Marisa Cunha, Filipa Fonseca-Dias, Ricardo Marinho, Marco Sampaio, Aníbal Marinho

**Affiliations:** 1 Intensive Care Unit, Unidade Local de Saúde de Santo António (ULSSA), Oporto, PRT; 2 Immunohemotherapy, Unidade Local de Saúde de Santo António (ULSSA), Oporto, PRT

**Keywords:** adverse drug reaction, agranulocytosis, case report, drug-induced neutropenia, metamizole

## Abstract

Agranulocytosis is a severe haematological disorder with multiple aetiologies, among which drug-induced causes are clinically significant. Metamizole is a non-opioid analgesic and antipyretic agent, used for the management of severe pain and high fever unresponsive to other measures. However, its use remains controversial due to the risk of metamizole-induced agranulocytosis (MIA), a rare but potentially life-threatening adverse reaction. This case aims to raise clinical awareness of MIA, highlighting the importance of its cautious use in daily practice. To our knowledge, it provides valuable clinical insight given the rapid onset of severe agranulocytosis, absence of alternative causative factors, and successful recovery with early intervention.

We present the case of a 70-year-old female patient who underwent elective abdominoplasty. Post-operative period was complicated with abdominal pain and sustained fever due to an abscess on the abdominal wall. Initial management with paracetamol and empiric antibiotics was ineffective. After surgical drainage, fever persisted and treatment with metamizole was started. Five days after starting metamizole, laboratory tests revealed agranulocytosis (90/µL). Metamizole was discontinued, and patient was placed under protective isolation. She received granulocyte-colony stimulating factor (G-CSF) and broad-spectrum antibiotics. Diagnostic workup excluded other sources of infection and neoplastic causes of agranulocytosis. Clinical and laboratory findings suggested MIA. Patient completed three days of G-CSF and 30 days of antibiotics, achieving full clinical and haematological recovery, with no recurrence during follow-up.

Metamizole-induced agranulocytosis is a rare but serious adverse reaction requiring early recognition. This case illustrates the importance of maintaining clinical vigilance and performing early haematological monitoring, particularly in high-risk patients. Clinicians should exercise caution when prescribing metamizole, limiting its use to well-justified indications and short durations. Continuous pharmacovigilance and further epidemiological studies are essential to define the true incidence, risk factors, and optimal management strategies.

## Introduction

Agranulocytosis is a severe haematological disorder defined as a peripheral neutrophil count of less than 500/µL, in the absence of significant anaemia and thrombocytopaenia, predisposing patients to life-threatening conditions [1,2]. 

In the current era of medicine, agranulocytosis has become an uncommon occurrence, with an incidence of 1.1 to 4.9 cases per million cases per year and an estimated mortality rate of 5% [3]. Although its pathogenesis is not fully understood, advanced age, female sex, and autoimmune disease are recognized risk factors. Neutrophil count < 100/µL, concomitant infection or shock, and pre-existing comorbidities, such as renal failure, are linked to worse outcomes [4].

Agranulocytosis can result from various causes, including drug-induced reactions, bone marrow failure, autoimmune diseases, infections, and nutritional deficiencies. Drug-induced cases are clinically significant, often due to direct marrow suppression or immune-mediated mechanisms [1,4]. Metamizole (dipyrone), a pyrazolone derivative with analgesic, antipyretic, and spasmolytic effects, is used for severe pain and high fever unresponsive to other therapies [2,5]. Despite its renewed use, metamizole remains controversial due to its potential to cause acute, potentially life-threatening agranulocytosis [1,2,4,6].

While the pathophysiology of metamizole-induced agranulocytosis (MIA) is not yet fully understood, it is thought to involve either immune-mediated neutrophil destruction via drug-dependent antibodies or interference with granulopoiesis by toxic metabolites [4]. Advanced age, female sex, and autoimmune disease are recognised risk factors [1,4].

The incidence and risk of MIA remain uncertain [1]. Safety concerns have led to heterogeneous recommendations, variable accessibility, and regulatory restrictions across countries. While metamizole remains available in several European Union member states, it has been withdrawn or never approved in many others [6,7]. Pharmacovigilance data from the EudraVigilance database report 1,448 suspected cases of MIA over approximately 30 years across 31 countries, predominantly from Germany, Spain, and Switzerland [8]. Agranulocytosis due to metamizole is a well-established adverse reaction. Although considered rare, its true incidence in clinical practice is likely underestimated, as pharmacovigilance data are subject to underreporting [1,4].

We report the case of a 70-year-old patient who developed agranulocytosis following metamizole treatment. Although other potential risk factors for agranulocytosis were present, the patient’s neutrophil count decreased to 90/µL only after exposure to metamizole. MIA was suspected. Patient was placed under protective isolation and started treatment with granulocyte-colony stimulating factor (G-CSF). Along with strict avoidance of metamizole, neutrophil count steadily increased to normal range of values. There was no evidence of recurrent agranulocytosis.

This case aims to raise clinical awareness of MIA, highlighting the importance of using metamizole properly in daily practice.

## Case presentation

A 70-year-old female patient, with a past medical history of type 2 diabetes mellitus, arterial hypertension and dyslipidaemia, was admitted into the Plastic Surgery ward after an elective abdominoplasty, which proceeded uneventfully. On post-operative day five, patient developed diffuse abdominal pain and sustained fever (tympanic temperature 39ºC). Physical examination was otherwise unremarkable and patient denied any other symptoms.

**Figure 1:**
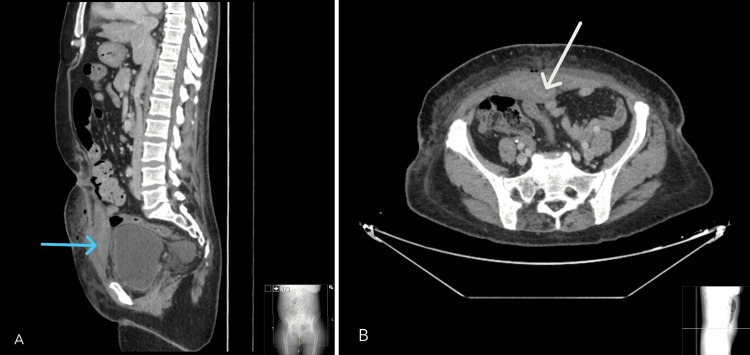
Abdominal CT scan: sagittal view (A) shows a fluid collection arising from the right rectus abdominis muscle, consistent with a hematoma (blue arrow), and demonstrating a partially organized wall suggesting abscess formation (axial view (B), white arrow). CT - computed tomography

**Figure 2:**
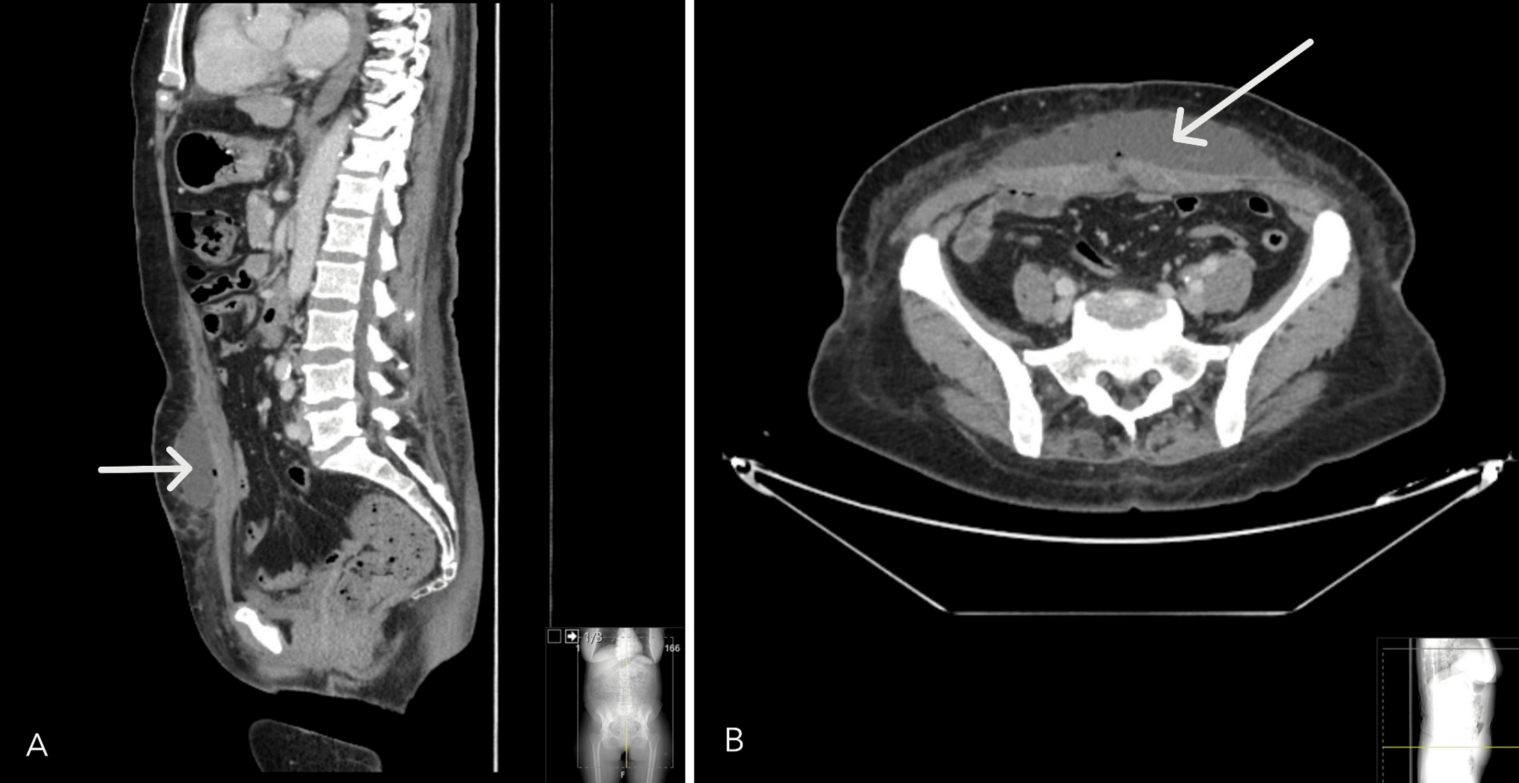
Abdominal CT scan: sagittal (A) and axial (B) views demonstrate a collection within the subcutaneous tissue of the anterior abdominal wall, containing fluid and gas, suggestive of an abscess (white arrows). CT - computed tomography

As presented in Table 1, laboratory findings revealed neutrophilia along with elevated C-reactive protein. At that time, blood and urine specimens are obtained for microbiological analysis. Paracetamol and empiric antibiotic therapy with amoxicillin and clavulanic acid were initiated, but with no response. On the sixth day following admission, an abdominal computed tomography (CT) was performed, revealing a suspected abscess in the right rectus abdominismuscle wall (Figure 1). Empirical antibiotic therapy was switched to piperacillin/tazobactam. A follow-up CT scan obtained one week later demonstrated complete resolution of the prior collection, with a new collection noted in the anterior abdominal wall (Figure 2). The abscess was managed with surgical drainage and piperacillin/tazobactam was maintained.

**Table 1:** Timeline values of the clinical case D – day; G-CSF - Granulocyte-colony stimulating factor; HA – Hospital admission; MTZ – Metamizole; NR – Normal range

	D0 HA	D5	D1 MTZ	D5 MTZ	D3 G-CSF	Discharge	Follow-up	Values NR
Hemoglobin (g/dL)	12.2	11.5	11.7	11.8	11.8	11.7	13.5	12-15
Leukocytes (x10^3/µL)	10.26	15.60	8.65	1.21	5.35	21.10	5.11	4.00-11.00
Neutrophils (x10^3/µL)	7.60	12.70	5.91	0.09	1.58	14.26	2.75	2.00-7.50
Lymphocytes (x10^3/µL)	1.70	1.51	1.51	1.02	1.58	3.92	1.69	1.50-4.00
Monocytes (x10^3/µL)	0.61	0.82	0.59	0.01	0.05	0.11	0.42	0.20-0.80
Eosinophils (x10^3/µL)	0.39	0.40	0.35	0.02	1.00	2.47	0.25	0.04-0.40
Basophils (x10^3/µL)	0.06	0.06	0.06	0.00	0.11	0.19	0.08	0.02-0.10
Platelets (x10^3/µL)	254	335	398	214	278	321	309	150-400
C-reactive protein (mg/L)	1.21	194.22	53.82	77.22	85.39	73.62	1.94	<5.00

Despite therapy, patient remained febrile and started on daily intravenous metamizole, one gram administered every eight hours. Five days later, laboratory tests showed leukopenia *de novo* characterized by a significant reduction in neutrophils (Table 1), establishing a diagnosis of agranulocytosis. Considering the increased risk of infection, she was placed under protective isolation and metamizole was immediately discontinued.

Patient was hemodynamically stable and reported no other symptoms. Physical examination revealed no rash, lymphadenopathy, or signs of pharyngitis. Abdominal examination, pulmonary and cardiac auscultation were unremarkable. Chest radiography showed no abnormalities, and aside from persistent fever and abnormal blood counts, no other clinical signs or symptoms were present. Earlier culture tests showed no growth.

A follow-up abdominal CT scan demonstrated a favorable evolution of the previously observed findings, with no other radiological evidence of infection. Treatment with G-CSF together with empiric intravenous antibiotic therapy with imipenem was initiated.

Peripheral blood smear confirmed neutropenia and revealed rarely stimulated lymphocytes with no blasts or other immature cells. Myeloid precursors were identified (approximately 10%), consistent with G-CSF treatment. Lymphocyte immunophenotyping revealed no features of lymphoproliferative disease. No neoplastic causes of agranulocytosis were found.

During diagnostic workup, no other sources of infection were found. Repeated blood cultures and urinalysis were obtained yielding negative results. Serology for Epstein-Barr virus (EBV), cytomegalovirus (CMV), hepatitis B (HBV), hepatitis C (HCV), and human immunodeficiency virus (HIV) showed no evidence of acute infection. A SARS-CoV-2 test was also negative. No evidence of nutritional deficiency was identified as the cause of agranulocytosis, with folate and vitamin B12 concentrations within normal limits.

Available pharmacy records were reviewed, and a structured, comprehensive clinical interview was conducted with emphasis on recent drug exposure, timing of administration, and corresponding laboratory abnormalities. This systematic assessment strengthened the suspicion of MIA.

Patient was assessed by an HLH-specialized team in order to exclude hemophagocytic lymphohistiocytosis (HLH). A cytokine storm was deemed unlikely, with an HScore of 132 (9.9% probability). Drug-induced agranulocytosis - most plausibly attributed to metamizole - was determined to be the most likely etiology.

Throughout hospitalization, patient remained hemodynamically stable, received G-CSF for three days, and completed a 30-day course of empiric antibiotic therapy, resulting in sustained clinical improvement. With these measures, alongside strict discontinuation of metamizole, her neutrophil count progressively recovered to normal levels.

Patient was discharged in an asymptomatic state, with no recurrence of agranulocytosis noted during follow-up. She was advised to avoid any future exposure to metamizole. Blood count timeline values can be seen in Table 1.

## Discussion

Metamizole-induced agranulocytosis remains a subject of ongoing debate. Its risk factors are not clearly defined; clinical presentation is often non-specific, and the underlying pathophysiological mechanisms are not yet fully understood. Furthermore, no reliable diagnostic tools exist to confirm causality with certainty. Reported incidence varies widely, contributing to persistent uncertainty regarding the true risk of MIA.

We present the case of a 70-year-old woman in whom MIA was suspected, underlining the need for high level of suspicion of this diagnosis. While alternative causes of agranulocytosis, most notably infection, were contemplated, the temporal association with metamizole exposure was compelling, as the drug had been initiated for persistent fever, an approved indication. Discontinuation of metamizole administration, combined with supportive therapy, led to clinical improvement and restoration of the neutrophil count.

The true incidence of MIA remains uncertain, with reported rates ranging from 0.56 to 0.96 cases per million inhabitants per year [2,9]. More recent data from a German matched-control cohort suggest a higher risk, estimated at approximately one in 1,602 patients treated with metamizole [10]. These differences likely reflect methodological variability and substantial under-reporting inherent to pharmacovigilance systems, as well as country-specific differences in regulatory status and prescribing practices [4,8].

Comparing prescription volumes with documented cases may provide a more accurate estimate of the true incidence of MIA. Although dose and duration of metamizole exposure have been proposed as potential contributors, current evidence suggests that MIA is largely idiosyncratic and not dose-dependent, reflecting underlying immunological and metabolic susceptibility factors [2,4,8,9]. Prolonged treatment, however, appears to increase the risk of agranulocytosis [9]. While no definitive latency period has been established, most reports describe onset within seven to 14 days after treatment initiation [7,8]. In the EudraVigilance database, the median time from metamizole initiation to agranulocytosis onset was 13 days, with a notably shorter interval in previously exposed patients [8].

In the present case, agranulocytosis developed within less than one week, earlier than typically reported. Previous sensitisation could theoretically account for this rapid presentation. A structured and detailed medication history was obtained through clinical interview and review of pharmacy records, and patient denied prior exposure to metamizole. However, given its broad over-the-counter availability in our country, unrecorded prior exposure cannot be entirely excluded, and such sensitisation may explain the unusually rapid onset observed. Notably, MIA may develop at any point during therapy and even after discontinuation [1,8]. Although it is generally considered a non-dose-dependent reaction, the possibility that higher doses or prolonged exposure may still facilitate sensitisation cannot be entirely excluded.

Several potential risk factors have been associated with MIA, although predictors and triggers remain a matter of debate. Reported risk factors for MIA include previous leukopenia, HCV infection, drug hypersensitivity, autoimmune diseases, and concomitant therapies, particularly methotrexate [8]. Female sex and older age have also been consistently identified as susceptibility factors [1,4]. However, a German series analysing 161 cases did not confirm specific predictors [7]. Older age, infection or sepsis, concomitant methotrexate therapy, and pancytopenia have been associated with an increased risk of severe complications and mortality [7].

Our patient's past medical history was unremarkable for any history of allergies, autoimmune disease, prior adverse drug reactions, or relevant co-medication. However, her demographic profile (older female) aligns with recognised risk characteristics [1,4]. This case highlights the importance of identifying patients with potential susceptibility to ensure prudent use of metamizole and appropriate monitoring for agranulocytosis.

Metamizole-induced agranulocytosis may present with a wide spectrum of clinical manifestations, often nonspecific. Patients may be asymptomatic or present with fever, sore throat, or general malaise. More suggestive of MIA is the combination of systemic symptoms with mucosal inflammation, such as aphthous stomatitis, pharyngitis, tonsillitis, or proctitis, which may progress to ulceration as the condition evolves [1,10]. Fever was the only presenting symptom in our patient. Given this nonspecific presentation, clinicians should maintain a high index of suspicion for MIA, particularly in patients recently exposed to metamizole.

Most cases are asymptomatic or only mildly symptomatic and therefore detected incidentally through routine blood tests. In the absence of a specific diagnostic test for drug-induced agranulocytosis, careful exclusion of alternative causes is essential. Further diagnostic workup was performed and no other source of infection was identified, nor any underlying neoplastic process. 

Although beta-lactam antibiotics are a recognized, albeit rare, cause of agranulocytosis, particularly with prolonged therapy, their contribution was assessed. Using the Naranjo causality scale [11], combined with the strong temporal association and the patient’s recovery after drug withdrawal, metamizole remained the most plausible causative agent.

Besides diagnostic workup, a detailed clinical history is crucial to identify predisposing factors and to determine which patients should avoid metamizole therapy [1]. Several risk factors, including older age, female sex, and concomitant infection, were present.

The management of MIA remains challenging, as standardized clinical recommendations have not yet been established. Available data strongly supports immediate discontinuation of metamizole and other potential offending agents, prompt initiation of broad-spectrum antibiotic therapy, administration of G-CSF, protective isolation, and close laboratory monitoring [1,4,7].

Our patient was receiving 1 gram of metamizole three times daily, a dose and frequency in agreement with commonly recommended therapeutic ranges for adult post-operative analgesia and antipyresis [12]. On the fifth day of metamizole therapy, agranulocytosis was detected incidentally through routine blood testing. Metamizole was promptly discontinued, and due to an absolute neutrophil count of 90/µL, G-CSF therapy was initiated. Broad-spectrum antibiotics were maintained due to persistent fever and the patient’s high risk of bacterial complications during profound neutropenia.

A comprehensive diagnostic evaluation excluded alternative etiologies of agranulocytosis, and serial blood counts were closely monitored throughout hospitalization. No definitive evidence-based guidelines exist regarding routine hematologic monitoring. Current literature supports, and the authors concur with, regular blood count surveillance, particularly during the first weeks of therapy [1].

Such timely interventions may facilitate earlier detection of MIA and improve clinical outcomes, especially in patients with identifiable risk factors. The EudraVigilance database reports a mortality rate of approximately 16% in MIA [8]; however, more recent data suggest a decline in fatality, likely reflecting heightened clinical awareness, earlier detection, and rapid drug withdrawal combined with antimicrobial therapy and/or G-CSF support [1]. These observations emphasize the critical importance of clinical suspicion and early therapeutic action, even in the absence of evidence-based protocols.

These findings must, however, be interpreted with caution. As with all single-case reports, causality is inferred primarily from temporal association and clinical judgment rather than from definitive diagnostic testing, as no standardized biomarkers for MIA currently exist. Furthermore, the potential influence of undetected confounding factors cannot be excluded, and the generalizability of this observation remains inherently limited. Underreporting and inconsistencies in pharmacovigilance data add further complexity to the accurate estimation of true incidence and risk profiles. Indeed, much of the current literature on MIA derives from spontaneous case reports within pharmacovigilance systems. Although valuable for detecting rare adverse reactions, such data are inherently limited and subject to biases that affect risk estimation [1,4,8]. Large, well-designed controlled studies are therefore needed to generate more robust epidemiological and clinical data.

This case contributes to the existing body of evidence by highlighting that even very rare adverse reactions can occur in routine clinical practice, underscoring the need to minimize the risk of agranulocytosis. To this end, it is essential that healthcare professionals prescribe metamizole only when clearly indicated, avoid exceeding the recommended duration of therapy, and carefully assess the risk-benefit balance prior to its use. Following the initiation of metamizole, laboratorial and clinical vigilance for early symptoms is crucial.

## Conclusions

Metamizole-induced agranulocytosis remains a rare but potentially fatal adverse reaction that is often underrecognized. This case underscores the diagnostic challenges posed by its nonspecific presentation and unpredictable onset, highlighting the importance of maintaining a high index of suspicion. Early identification, prompt discontinuation of metamizole, close hematological monitoring, and timely supportive care are essential for improving outcomes.

While a single report cannot establish practice standards, it reinforces the need for cautious prescribing, limiting metamizole use to well-justified indications and the shortest effective duration. Ongoing pharmacovigilance, systematic case reporting, and large-scale studies are needed to better define the true incidence, risk factors, and optimal management strategies for this potentially life-threatening condition.
